# Psychosocial stress and cortisol stress reactivity predict breast milk composition

**DOI:** 10.1038/s41598-021-90980-3

**Published:** 2021-06-02

**Authors:** Anna Ziomkiewicz, Magdalena Babiszewska, Anna Apanasewicz, Magdalena Piosek, Patrycja Wychowaniec, Agnieszka Cierniak, Olga Barbarska, Marek Szołtysik, Dariusz Danel, Szymon Wichary

**Affiliations:** 1 Institute of Zoology and Biomedical Research, Jagiellonian University, Gronostajowa 9, 30-387, Kraków, Poland; 2 Ludwik Hirszfeld Institute of Immunology and Experimental Therapy, Polish Academy of Sciences, Rudolfa Weigla 12, 53-114 Wroclaw, Poland; 3 Institute of Psychology, University of Wroclaw, J.W. Dawida 1, 50-527 Wroclaw, Poland; 4 Faculty of Biology, Department of Human Biology, University of Wroclaw, Szewska 48, 50-139 Wroclaw, Poland; 5 Faculty of Medicine and Health Sciences, Andrzej Frycz Modrzewski Krakow University, Gustawa Herlinga-Grudzinskiego 1, 30-705 Krakow, Poland; 6 Laboratory of Human Milk and Lactation Research At Regional Human Milk Bank in Holy Family Hospital, Faculty of Health Sciences, Medical University of Warsaw, Zwirki i Wigury 63a, 02-091 Warsaw, Poland; 7 Faculty of Biotechnology and Food Science, Wroclaw University of Environmental and Life Sciences, J. Chelmonskiego 37, 51-630 Wrocław, Poland; 8 Institute of Psychology, Jagiellonian University, Ingardena 6, 30-060 Krakow, Poland

**Keywords:** Stress and resilience, Nutrition, Paediatric research

## Abstract

We studied a sample of 146 Polish, exclusively breastfeeding mothers and their healthy born on time infants to explore the effect of perinatal psychosocial stress on breast milk composition. Maternal perinatal stress was assessed using Recent Life Changes Questionnaire summarizing stressful events from the previous six months. Stress reactivity was determined by administering the cold pressor test and measuring cortisol in saliva samples taken during the test. Breast milk sample was taken to measure energy, protein, fat, lactose, and fatty acid content. Analyses revealed that stress reactivity was positively associated with milk fat and long-chain unsaturated fatty acids and negatively associated with milk lactose. Perinatal psychosocial stress negatively affected energy density, fat as well as medium-chain and long-chain saturated fatty acids in milk. These results, together with previous studies, advocate monitoring maternal psychological status during the peripartum to promote breastfeeding and healthy infant nutrition.

## Introduction

Psychosocial stress is a potent lifestyle factor with the ability to affect many biological processes, including reproduction^1,2^. Long-term and chronic coping with stress induces metabolic and behavioral effects, which require a considerable amount of energy ^3,4^. Because many body processes compete for access to limited energy resources, reproductive function is severely compromised when chronic stress occurs. The negative effect of psychosocial stressors and high stress reactivity was observed in various stages of the reproductive process in women, including lactation^5–8^.

Extensive evidence demonstrates that child and adult health risks, including obesity, hypertension, and diabetes, may be programmed by early life nutritional status^9^. Breastfeeding, which typically occurs during this period, is a critical first step to ensure best-tailored nutrition delivered by the mother to support child development and optimal health later in life. Failure to provide key macro- and micronutrients as well as beneficial breast milk microbiota results in early metabolic alterations with lifelong consequences for health. Understanding maternal factors that affect breastfeeding, as well as breast milk composition, is critical in this respect.

A recent study in the US demonstrated over 40% of breastfeeding women experiencing at least two major life events such as financial, emotional, and partner-related problems during the year preceding the birth of their child. The presence of these stressors was associated with a lower likelihood of initiation and a higher risk of early termination of breastfeeding^10^. The results of this study suggest that psychosocial stress is a potent factor with the ability to shape breastfeeding performance. However, the overall effect of psychosocial stress on lactation is poorly understood. The existing studies provide two kinds of evidence: (1) that there is little to no effect of stress on lactation due to the ability of the hormonal milieu accompanying lactation to alleviate the physiological response to psychosocial stressors^11^; or (2) that psychosocial stressors may negatively affect breastfeeding via the hormonal stress response^5^. While a considerable number of studies demonstrated the negative effect of stress on breastfeeding initiation, duration, and exclusivity, only a few studies examined the impact of stress on breast milk composition^12^.

Psychosocial stress may affect the composition of breast milk via several pathways. Direct pathways involve the short-term effect of cortisol, which increases in response to psychological challenges and regulates the metabolism of glucose and lipids. Experimental studies in which subjects were administered hydrocortisone demonstrated a steady but transient increase of glucose and free fatty acids in plasma and serum shortly after infusion^13,14^. Thus, cortisol rising in response to short-term stressors in breastfeeding mothers may increase the level of substrate necessary for carbohydrate and lipid synthesis and, consequently, result in higher lactose and fat levels in milk.

While acute stress may have only a limited effect on breast milk composition, chronic stress might incur more pronounced consequences due to its overall effect on metabolism. Indirect pathways may include effects on dietary intake in response to long-term stressors. Studies evidence both the increase and the decrease in the consumption of energy-dense food during periods of high perceived stress^15–17^. Since the fat composition of human milk is highly dependent on the maternal diet, changes in energy and dietary fat intake under stress may result in changes in fat and fatty acid components of breast milk.

Moreover, chronic stress affects body composition by increasing fat accumulation^17^. This may result in a higher concentration of lipids and specific fatty acids in breast milk, because milk fat synthesis partly relies on maternal fat resource^18^. By increasing fat accumulation, chronic stress may also promote insulin resistance and heighten the risk of gestational diabetes. Previous studies in insulin-resistant and diabetic mothers demonstrated the altered composition of breast milk, primarily in energy, lipids, and fatty acids^19,20^.

Physiological mechanisms described above suggest that maternal psychological stress not only influences breastfeeding initiation and duration but also affects the composition of breast milk, yet evidence for such action is scarce. A recent review on the effect of postnatal anxiety on breastfeeding outcomes identified only four studies that address effects on breast milk composition^12^. These studies, however, focus only on the effect of maternal anxiety on the immunologically active components of breast milk, cortisol and sodium concentration, and have produced mixed results^21–24^. To our knowledge, none of the published studies have examined the potential of psychosocial stress to modify the content of milk macronutrients and the fatty acid profile. Thus, overall, the effect of psychological stress, which frequently accompanies childbirth and childcare, remains unclear.

The current study aimed to deepen the understanding of the effect of psychosocial stress by investigating how the stress associated with recent life changes as well as maternal cortisol reactivity affects macronutrients, energy density, and fatty acid levels in the milk of exclusively breastfeeding women. Taking into consideration the abovementioned physiological mechanisms and the fact that lipids are the most variable components of breast milk^25–29^, we hypothesized that long-term maternal stress would negatively affect the energy value of milk, the total content of carbohydrates and fats as well as fatty acids profile. Moreover, cortisol level, which influences lipid and carbohydrate metabolism and breast milk synthesis, should also be associated with the composition of breast milk. Specifically, higher cortisol reactivity would be related to lower carbohydrate content and higher lipid content in milk.

## Results

No differences in maternal and infant characteristics were found between groups of mothers with low vs. high recent life changes (RLC) except for the total cortisol level (Cort_AUC_) during the cold-pressor test (CPT), which was higher in high RLC mothers (*p* = 0.05). Levels of cortisol in high RLC mothers were also increased during the consecutive time points of CPT – 10 min before (Cort_0_) and immediately before CPT (Cort_10_), during the CPT (Cort_11_), and 10 min after the CPT (Cort_21_) (Table 1).

**Table 1:** General characteristics of 147 mothers and infants participating in the study and differences between lower and higher RLC (Recent Life Changes) score groups.

	All women N = 146 Mean (SE)	Low RLC N = 74 Mean (SE)	HighRLC N = 72 Mean (SE)
**Maternal**			
Age (years)	31.0 (0.31)	30.8 (0.43)	31.2 (0.46)
Education (% higher)	94.6	91.7	97.3
Life satisfaction	5.8 (0.08)	5.8 (0.11)	5.8 (0.11)
Economic satisfaction	5.5 (0.08)	5.4 (0.13)	5.6 (0.11)
Number of children	1.0 (1.0–2.0)	2.0 (1.0–2.0)	1.0 (1.0–2.0)
Body fat %	28.4 (0.55)	28.1 (0.77)	28.8 (0.85)
Cort_AUC_ (ng/ml)	6.4 (0.55)	*4.9 (0.48)	*7.9 (0.99)
Cort_0_ (ng/ml)	2.3 (0.19)	1.9 (0.22)	2.8 (0.33)
Cort_10_ (ng/ml)	2.2 (0.21)	1.7 (0.18)	2.8 (0.39)
Cort_11_ (ng/ml)	2.05 (0.19)	1.5 (0.15)	2.6 (0.35)
Cort_21_ (ng/ml)	1.9 (0.19)	1.5 (0.16)	2.4 (0.35)
RLC Score	401 (17.7)	**231 (10.5)	**573 (18.8)
**Infant**			
Age (Months)	4.7 (0.04)	4.7 (0.06)	4.7 (0.07)
Sex (% of girls)	46.7	44.0	49.3
Weight-for-height at birth	-2.8 (0.10)	-2.8 (0.15)	-2.9 (0.14)
Weight-for-height at 5 mo	-0.7 (0.10)	-0.7 (0.13)	-0.6 (0.15)

*p < 0.05.

**p < 0.01.

Low RLC mothers produced milk with higher fat content (*p* = 0.01) and higher energy density (*p* = 0.01) than did high RLC mothers (Table 2). Milk produced by women from these two groups also differed with respect to the fatty acid profile (Table 3). Low RLC mothers produced milk with higher medium chain fatty acids (MCFA) (*p* = 0.004) and long chain fatty acids (LCFA) content (*p* = 0.02) (unsaturated and saturated) but did not differ significantly from the other group with respect to long chain polyunsaturated fatty acids (LC-PUFAs) content. The effect sizes were medium (Cohen’s d from *d* = 0.38 for all LCFAs to *d* = 0.48 for MCFAs).

**Table 2:** Nutritional components of breast milk samples collected from mothers participating in the study and differences between lower and higher RLC (Recent Life Changes) score groups.

	All women (N = 146) Mean (SE)	Lower RLC (N = 74) Mean (SE)	Higher RLC (N = 72) Mean (SE)
Fat (g/100 ml)	4.2 (0.11)	4.5 (0.17)	3.9 (0.15)
Proteins (g/100 ml)	0.8 (0.01)	0.8 (0.02)	0.8 (0.01)
Lactose (g/100 ml)	7.2 (0.02)	7.2 (0.03)	7.2 (0.03)
Energy (kcal/100 ml)	71.7 (1.05)	74.3(1.55)	69.1 (1.38)

**Table 3:** Fatty acids of breast milk samples collected from mothers participating in the study and differences between lower and higher RLC score groups. MCFA (medium chain fatty acids), LCFA (long chain fatty acids), LCFA-S (long chain saturated fatty acids), MUFA (monounsaturated fatty acids), PUFA (polyunsaturated fatty acids).

	All women (N = 146) Mean (SE)		Lower RLC (N = 74) Mean (SE)		Higher RLC (N = 72) Mean (SE)	
	g/100 g fat	g/100 ml milk	g/100 g fat	g/100 ml milk	g/100 g fat	g/100 ml milk
C10:0	2.1 (0.06)	0.09 (0.004)	2.2 (0.08)	**0.10 (0.006)	2.0 (0.09)	**0.08 (0.005)
C12:0	2.8 (0.05)	0.12 (0.004)	2.9 (0.08)	**0.13 (0.006)	2.7 (0.07)	**0.11 (0.005)
C14:0	3.4 (0.05)	0.14 (0.005)	3.4 (0.07)	0.15 (0.007)	3.4 (0.08)	0.14 (0.006)
C15:0	0.2 (0.01)	0.01 (0.001)	0.2 (0.02)	0.01 (0.001)	0.2 (0.02)	0.009 (0.0006)
C16:0	19.1 (0.17)	0.81 (0.020)	19.3 (0.20)	**0.87 (0.03)	18.9 (0.28)	**0.75 (0.03)
C18:0	5.8 (0.08)	0.24 (0.007)	5.8 (0.11)	*0.26 (0.01)	5.8 (0.11)	*0.23 (0.01)
C18:2n6 (LA)	10.7 (0.24)	0.44 (0.02)	*10.1 (0.32)	0.45 (0.02)	*11.1 (0.36)	0.43 (0.020)
C18:3n3 (ALA)	2.0 (0.08)	0.08 (0.004)	1.9 (0.12)	0.09 (0.007)	2.0 (0.12)	0.08 (0.005)
C20:4n6 (AA)	0.4 (0.03)	0.02 (0.001)	0.3 (0.04)	0.02 (0.002)	0.4 (0.05)	0.02 (0.002)
C20:5n3 (EPA)	0.2 (0.02)	0.006 (0.001)	0.2 (0.03)	0.007 (0.001)	0.1 (0.04)	0.006 (0.001)
C22:6n3 (DHA)	0.15 (0.024)	0.006 (0.001)	0.13 (0.033)	0.006 (0.002)	0.17 (0.036)	0.006 (0.001)
MCFA	5.0 (0.11)	0.21 (0.008)	5.2 (0.14)	**0.23 (0.010)	4.8 (0.16)	**0.19 (0.010)
LCFA	83.7 (0.33)	3.51 (0.100)	83.2 (0.46)	*3.74 (0.140)	84.1 (0.48)	*3.30 (0.130)
LCFA-S	28.6 (0.24)	1.20 (0.030)	28.8 (0.29)	*1.29 (0.050)	28.4 (0.38)	*1.12 (0.050)
MUFA	55.2 (0.37)	2.31 (0.06)	41.8 (0.27)	**1.89 (0.070)	42.0 (0.34)	**1.63 (0.066)
PUFA	13.3 (0.34)	0.55 (0.020)	12.6 (0.44)	0.57 (0.030)	13.9 (0.51)	0.54 (0.030)

*p < 0.05.

**p < 0.01.

### RLC, maternal diet, body fat and cortisol reactivity

A positive correlation was found between maternal RLC and log-transformed Cort_AUC_ (logCort_AUC_) during the CPT (*r* = 0.2*; p* = 0.02). This association was independent of infant age, which was found to be negatively related to logCort_AUC_ (*r* = -0.2*, p* = 0.02), maternal age and body fat content. In addition, maternal RLC correlated positively with cortisol levels in each of the consecutive time points of CPT test (*r* = 0.19*, p* = 0.02 for Cort_0_, Cort_10_, and Cort_11_ and *r* = 0.22*, p* < 0.01 for Cort_21_).

No associations were found between RLC and maternal diet. In contrast, positive associations were found between maternal body fat and fat consumption. Notably, higher maternal body fat was associated with higher intake of total fat and MCFAs as well as LC-SFAs (Table 4).

**Table 4:** Correlation between maternal body fat, dietary intake of nutritional components, maternal stress (RLC), and cortisol level (Cort_AUC_).

	RLC	logCort_AUC_	Body fat%
Body fat %	0.02	0.08	-
Energy	0.11	0.10	0.6
Protein	0.15	0.14	0.8
Fat	0,01	0.02	0.18*
Carbohydrate	0.11	0.10	0.10
MCFA	−0.04	0.04	0.22**
LCFA	0.004	−0.004	0.17*
LCFA-S	0.02	−0.004	0.23**
MUFA	−0.05	−0.008	0.14
PUFA	0.03	0.007	0.03

*p < 0.05.

**p < 0.01.

### RLC, cortisol and breast milk macronutrients

**Figure 1:**
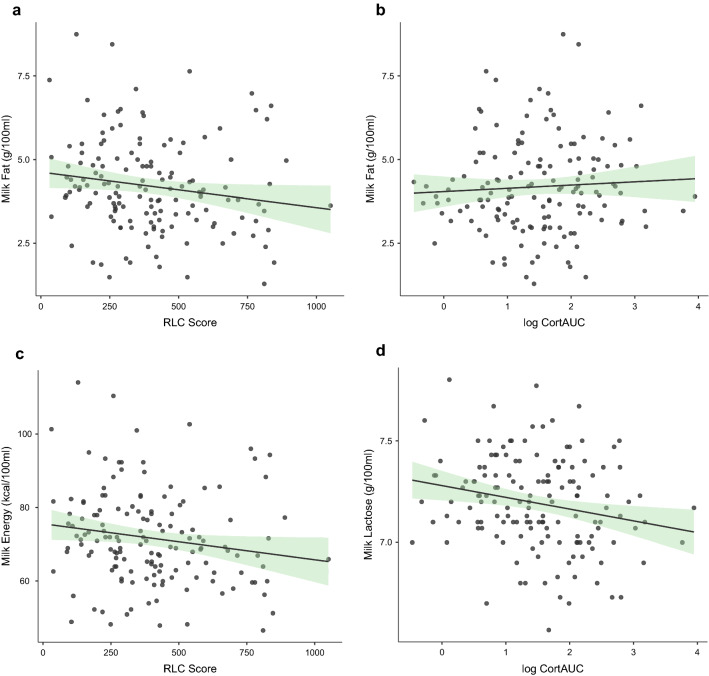
Recent Maternal stress (RLC) and cortisol levels are associated with the level of nutrients in breast milk. Higher maternal stress (RLC) is associated with lower levels of fat (panel **a**) and energy (panel **c**) in milk. Higher maternal cortisol is associated with higher fat (panel **b**) and lower lactose (panel **d**) in milk.

RLC and logCort_AUC_ were both associated with the content of the main nutritional components of the mothers' milk (Table 5). Specifically, RLC was negatively (p = 0.048), while logCort_AUC_ was positively (*p* = 0.045) associated with fat content (Fig. 1A,B). Common factors included in the null model—maternal body fat, child age, child BMI, average number of feedings per day, and dietary intake of fat—together explained about 11% of the variance in the content of fat in milk. Adding postnatal RLC and logCort_AUC_ increased the variance explained by the model to about 14%.

**Table 5:** Maternal stress (RLC) and total cortisol level (logCort_AUC_) predict the main nutrients of breast milk. Significant associations (p < 0.05) between maternal stress and cortisol levels were found for concentrations of milk fat, lactose, and energy in milk samples collected from mothers.

Model	Adjusted R2	p	R2 change Model 0 to 1	p	β	p
**Milk fat**						
Model 0	0.11	< 0.001				
Child age					0.01	0.89
Child BMI					− 0.02	0.77
Body fat%					0.16	0.06
Dietary fats					0.05	0.53
Number of feedings					− 0.34	< 0.001
Model 1	0.14	< 0.001	0.04	0.03		
Child age					0.05	0.56
Child BMI					− 0.01	0.88
Body fat%					0.16	0.05
Dietary fats					0.05	0.52
Number of feedings					− 0.36	< 0.001
RLC					− 0.16	0.04
logCort_AUC_					0.17	0.04
**Milk lactose**						
Model 0	< 0.01	0.33				
Child age					0.01	0.87
Child BMI					0.08	0.35
Body fat %					− 0.03	0.69
Dietary carbohydrates					− 0.16	0.06
Number of feedings					0.10	0.25
Model 1	0.05	0.04	0.06	0.01		
Child age					− 0.03	0.73
Child BMI					0.08	0.35
Body fat %					− 0.02	0.85
Dietary carbohydrate					− 0.12	0.15
Number of feedings					0.16	0.07
RLC					− 0.10	0.23
logCort_AUC_					− 0.22	0.01
**Milk protein**						
Model 0	-0.01	0.83				
Child age					0.07	0.44
Child BMI					− 0.05	0.57
Body fat %					0.11	0.22
Dietary protein					0.02	0.82
Number of feedings					− 0.03	0.70
Model 1	-0.02	0.75	0.01	0.35		
Child age					0.08	0.34
Child BMI					− 0.04	0.64
Maternal BMI					0.10	0.23
Dietary protein					0.04	0.67
Number of feedings					− 0.04	0.66
RLC					− 0.11	0.21
logCort_AUC_					0.08	0.37
**Milk energy**						
Model 0	0.10	0.002				
Child age					0.03	0.73
Child BMI					− 0.02	0.75
Body fat %					0.17	0.03
Dietary energy					− 0.05	0.50
Number of feedings					− 0.32	< 0.001
Model 1	0.12	< 0.001	0.03	0.06		
Child age					0.07	0.49
Child BMI					− 0.02	0.87
Body fat %					0.18	0.03
Dietary energy					− 0.04	0.55
Number of feedings					− 0.34	< 0.001
RLC					− 0.15	0.06
logCort_AUC_					0.17	0.09

The association with milk lactose content remained significant only for logCortAUC (Fig. 1C). Higher logCort_AUC_ was associated with lower carbohydrate content (*p* = 0.01), while RLC was not related to the level of carbohydrate. Common factors included in the null model did not predict the content of carbohydrates in milk. Adding RLC and logCort_AUC_ increased the statistical significance of the model and explained about 6% of the variance in the content of carbohydrates in milk.

RLC and logCort_AUC_ were also associated with milk energy density. For RLC, the association was negative (marginal significance, *p* = 0.06) (Fig. 1D), while for logCort_AUC_ it was positive, although not statistically significant. Overall, the final model explained about 12% of the variance with 3% explained by stress-related variables. No association was found between RLC and logCort_AUC_ and the milk protein content. None of the predictors included in the null and final model explained the variance in the protein content.

### RLC, cortisol and breast milk fatty acids

**Figure 2:**
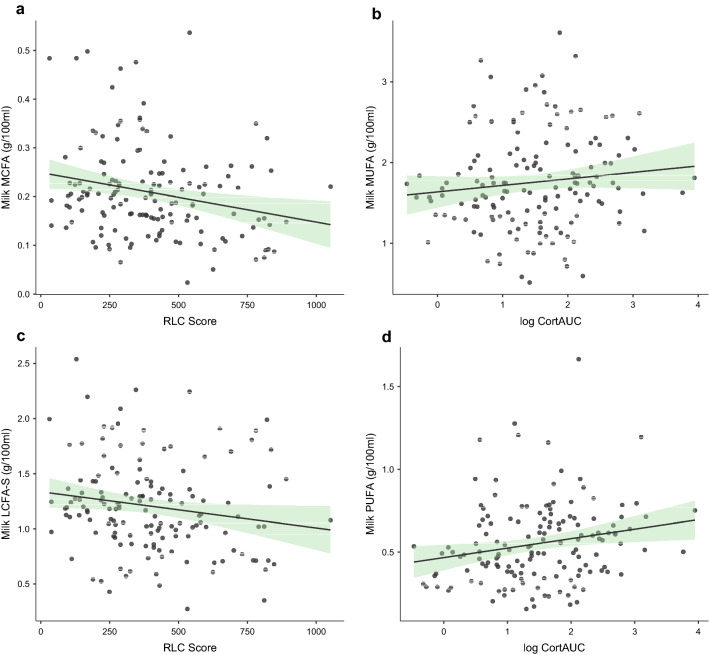
Maternal stress (RLC) and cortisol levels are associated with the level of fatty acids in milk. Higher maternal stress (RLC) is associated with lower levels of MCFA (panel **a**) and LCFA-S (panel **c**). Higher level of maternal cortisol is associated with higher levels of MUFA (panel **b**) and PUFA (panel **d**).

Postnatal stressors and stress reactivity were also related to the profile of milk fatty acids (Table 6). Higher RLC was associated with lower MCFA content (*p* = 0.01) (Fig. 2A), while logCort_AUC_ was not associated with it. Common factors included in the null model explained about 13% of the variance in the content of fat in milk. Adding RLC and logCort_AUC_ increased the variance explained by the model to about 16%.

**Table 6:** Maternal stress (RLC), cortisol level, and number of feedings predict fatty acid levels in breast milk. Significant associations (p < 0.05) between maternal stress and cortisol levels were found for concentrations of milk MCFA , LCFA, LCFA-S, MUFA, and PUFA.

Model	Adjusted R2	p	R2 change Model 0 to 1	p	β	p
**Milk MCFA**						
Model 0	0.13	< 0.001				
Child age					−0.03	0.74
Child BMI					−0.05	0.51
Body fat %					0.12	0.15
Dietary MCFA					−0.10	0.20
Number of feedings					−0.35	< 0.001
Model 1	0.16	< 0.001	0.04	0.04		
Child age					−0.01	0.86
Child BMI					−0.04	0.62
Body fat %					0.12	0.12
Dietary MCFA					−0.11	0.16
Number of feedings					−0.34	< 0.001
RLC					−0.20	0.01
logCort_AUC_					0.06	0.49
**Milk LCFA**						
Model 0						
Child age	0.10	0.001			< 0.01	0.96
Child BMI					− 0.04	0.65
Body fat %					0.14	0.09
Dietary LCFA					0.07	0.37
Number of feedings					− 0.33	< 0.001
Model 1	0.14	< 0.001	0.05	0.02		
Child age					0.05	0.56
Child BMI					− 0.02	0.78
Body fat %					0.14	0.09
Dietary LCFA					0.07	0.36
Number of feedings					− 0.36	< 0.001
RLC					− 0.13	0.09
logCort_AUC_					0.21	0.01
**Milk Milk LCFA-S**						
Model 0	0.12	< 0.001				
Child age					0.05	0.51
Child BMI					− 0.08	0.30
Body fat %					0.17	0.04
Dietary LCFA-S					< 0.01	0.97
Number of feedings					− 0.36	< 0.01
Model 1	0.15	< 0.001	0.03	0.06		
Child age					0.08	0.31
Child BMI					− 0.07	0.38
Body fat %					0.17	0.04
Dietary LCFA-S					< 0.01	0.98
Number of feedings					− 0.37	< 0.001
RLC					− 0.15	0.05
logCort_AUC_					0.14	0.08
**Milk MUFA**						
Model 0	0.10	0.001				
Child age					− 0.01	0.90
Child BMI					− 0.04	0.65
Body fat %					0.15	0.07
Dietary MUFA					< −0.01	0.99
Number of feedings					− 0.33	< 0.001
Model 1	0.15	< 0.001	0.05	0.01		
Child age					0.03	0.67
Child BMI					− 0.02	0.78
Body fat %					0.15	0.07
Dietary MUFA					< 0.01	0.98
Number of feedings					− 0.36	< 0.001
RLC					− 0.14	0.09
logCort_AUC_					0.22	0.009
**Milk PUFA**						
Model 0	0.11	< 0.001				
Child age					− 0.04	0.60
Child BMI					0.07	0.37
Body fat %					0.08	0.33
Dietary PUFA					0.32	< 0.001
Number of feedings					− 0.14	0.08
Model 1	0.15	< 0.001	0.05	0.01		
Child age					0.01	0.93
Child BMI					0.08	0.30
Body fat %					0.07	0.38
Dietary PUFA					0.32	< 0.001
Number of feedings					− 0.19	0.02
RLC					− 0.05	0.55
logCort_AUC_					0.25	0.003

RLC and logCort_AUC_ also predicted milk LCFA content. RLC was negatively associated with LCFA-S (*p* = 0.05) (Fig. 2C), while the positive association between logCort and LCFA-S was not significant (*p* = 0.08). Common factors included in the null model explained about 12% of the variance in the LCFA content, and adding RLC and logCort_AUC_ increased the variance explained by about 3%.

For MUFA, a significant and positive association was found for logCort_AUC_ (*p* < 0.01) (Fig. 2B) but not for RLC. Similarly, the PUFA content was positively affected only by logCortAUC (*p* < 0.01) (Fig. 2D) and was not related to RLC. These two factors together explained around 5% of the variance, while the whole final model explained about 15% of the variance in MUFA and PUFA.

## Discussion

The results of our study demonstrate a significant and negative effect of maternal stress during the postpartum period on the composition of breast milk. To our knowledge, for the first time, we report that the intensity and number of perceived stressors are negatively associated with the content of fat and energy density of breast milk. We also found that these perceived stressors were negatively associated with the content of medium-chain and long-chain saturated fatty acids. Finally, we found that the higher cortisol level secreted in response to a mild laboratory stressor was related to the higher fat and lower lactose content in milk. Cortisol level was also positively associated with the content of long-chain unsaturated fatty acids.

To explain these relationships, we propose that the increased level of cortisol released in response to a single maternal stressor acts to mobilize maternal fat resources and thus increases the pool of substrates available for fat synthesis in a short-term perspective. However, such mobilization of maternal resources, when associated with long-term stress will result in their gradual depletion. Thus, chronic stress will decrease the pool of substrates available for milk fat synthesis in a long-term perspective.

Experimental human studies demonstrated an increase in free fatty acids and glucose levels in serum that lasts for hours after the administration of cortisol or its analog hydrocortisone^13,14^. In the case of lactating mothers and milk synthesis, an increase in cortisol levels in response to short-term stress might be thus associated with higher accessibility of glucose and fatty acids for milk synthesis. However, responding to a stressor requires immediate energy utilization, and thus the reserves of glucose might be quickly depleted due to its primary role as the energy source for the brain during the stress response^42,3^. Although the energy needed for stress response might also be acquired from fatty acids, the mobilization and transport of fatty acids is relatively slow compared to glucose^43^.

As a consequence, as demonstrated in a study on metabolic changes during mental stress, there is a shift in the utilization of the substrate, from oxidizing mainly glucose at the beginning to oxidizing mainly lipids at the end of process^44^. Such a shift may result in differential access to the metabolic substrate for milk synthesis, leaving less substrate available for milk carbohydrate synthesis and more substrate for milk fat synthesis. Thus, a short-term increase in cortisol is associated with lower carbohydrate and higher fat content of milk, as demonstrated in our study. Partial support for this result comes from the study by Chen et al.^45^ who found lower milk lactose concentrations in breastfeeding mothers who had higher cortisol levels in cord blood.

In addition to the association between cortisol and milk macronutrients, we found that higher levels of cortisol were also associated with higher levels of total LC-FAs and LC-MUFAs and PUFAs. These relationships might be a consequence of a general action of glucocorticoids (GC) supporting fat tissue lipolysis during lactation. Studies on lactating animals (ruminants and rodents) confirm that lipolysis in fat tissue is stimulated by GC^46^. As a consequence, the use of adipose tissue-derived fatty acids influences the fatty acid composition of milk fat. Indeed, in dairy cattle and other animal species experiencing periods of negative energy balance during lactation, and the associated period of increased fat tissue lipolysis, the fatty acid composition of milk shows a marked increase in the proportion of long-chain fatty acids^47^. In addition to the indirect effect of GC on fat lipolysis, a study of in vitro mammary tissue of pregnant rabbit^48^ demonstrated a direct effect of GC on milk fatty acid synthesis. In that study, adding corticosterone to tissue cells cultured with insulin and prolactin was associated with an immediate increase in the synthesis of LCFAs. The presence of corticosterone in the culture was also associated with the increase in other groups of fatty acids (however, smaller than in the case of LCFAs), which may suggest an overall increase in milk fat production, also found in our study.

In contrast to the short-term action of cortisol, the postnatal life stress as indexed by RLC score depicts the cumulative effect of several stressors over the period of the last six months. The negative association of the RLC score with milk fat found in our study may reflect the gradual depletion of the substrates for milk fat synthesis under the influence of long-term stress. When physiological stress occurs during the energetically demanding process of lactation and is not balanced by the adequate maternal nutrient intake, it may result in the depletion of maternal energy resources accumulated in fat depots^49,50^.

Such an effect was demonstrated in Bangladeshi mothers who faced seasonal changes in food availability and in the level of energy expenditure^51^. Reaching the peak of lactation during the wet season, when food supply was low in relation to energy output, was associated with greater weight loss compared to when the same stage was reached during the dry season, when food supplies are more adequate. Seasonal changes and maternal fat resources were also found to correlate with breast milk fat content in African mothers living in similar environmental conditions^52^.

Although mothers from our study group did not suffer from restrictions in food availability, their overall food and fat intake remained unassociated with the number of life stressors or the cortisol level during laboratory stress (Table 4). Thus, the higher requirement for energy and nutritional substrates associated with stress was not balanced by the increase in maternal dietary intake. This could result in a gradual depletion of maternal fat resources and thus lower availability of substrates necessary for milk fat synthesis. It has to be acknowledged that while the assessed energy and nutrients intake covered only the most recent period, the RLC score summarized life stressors during the previous six months. However, one can argue that a large part of the observed variation in the RLC score may be attributed to stressors associated with childcare, which remained constant over time, including the recent period when dietary intake was assessed.

Our results support and elucidate the findings from two studies which examined the effectiveness of behavioral interventions to reduce psychological stress in breastfeeding mothers. Keith et al.^53^ found decreased milk production and decreased fat level in milk of mothers of preterm or critically ill infants from a control group with no support, compared to the other three groups with various forms of psychological support. The same effect was found in the study by Shukri et al.^54^, where mothers underwent an intervention to reduce the level of postpartum stress via listening to relaxing audio recordings. Mothers from the control group with no intervention had a higher level of stress and cortisol and lower levels of milk fat than the mothers from the intervention group.

The effect of chronic stress might be further evidenced by the association between the RLC score and the FA content in milk found in our study. Higher postnatal stress and lower maternal body fat predicted a lower amount of LCFA-S, while dietary intake of LCFA-S was not associated with it. Lack of the effect of dietary intake on milk LCFA-S levels suggests that nondietary sources of long-chain fatty acids, e.g., fat reserves, were utilized to support milk synthesis. Indeed, studies on lactating ruminants and rodents confirm that lipolysis of fat tissue rises in response to stimulation of the sympathetic nervous system and increased levels of catecholamines such as adrenaline and noradrenalin^47^. Furthermore, increased cortisol levels, as observed in our chronically stressed mothers, are associated with insulin resistance^55^, which was also demonstrated to stimulate lipolysis in lactating females^56^. Both the increased levels of catecholamines and cortisol constitute an integral part of the response to psychosocial stress and may contribute to the observed changes in milk content of LC-SFAs. Thus, repeating stressful events might be associated with more pronounced lipolysis and the subsequent loss of substrate necessary for milk fat and fatty acid synthesis.

Higher maternal stress was also associated with lower medium-chain fatty acid content in milk. Interestingly, the amount of MCFA in milk was not associated with cortisol levels, which may suggest that only the long-term stress is powerful enough to limit the pool of substrates necessary for milk MCFA synthesis. Indeed, rather than coming directly from maternal diet or fat storage, MCFA is synthesized by the mammary gland from a variety of substrate^57^. Thus, only the presence of chronic and long-term stressors might cause a decrease in the metabolic pool for synthesis. This effect might be consequence of the differences in FAs utilization under stress. As the energy source for tissue metabolism, triglycerides of MCFAs have several advantages over those containing more LCFAs^43^. First, they are more easily digested, and the liberated MCFAs are quickly absorbed in the intestinal lumen^58^. Second, in contrast to LCFAs, tissue metabolism of MCFAs does not depend on proteins for binding, transport, and transmembrane translocation. Therefore, they can serve as an immediately available source of energy, especially under conditions of stress^59^.

Finally, we found no association between the intensity and number of recent life stressors and the MUFAs and PUFAs. Previous studies in rats demonstrated that glucocorticoids and catecholamines are potent suppressors of the first desaturase reaction, which converts linoleic and alpha-linolenic acid to longer-chained FAs^60^. It was also demonstrated that omega-3 FAs act to reduce cortisol response to stressors in humans^61^. These observations imply that mothers may utilize omega-3 PUFAs to mitigate the effect of stress that might also be harmful to infants. However, it has to be acknowledged that none of the above studies was conducted in breastfeeding women that significantly differ from non-breastfeeding individuals with respect to the physiology of the stress response^11^.

On the other hand, studies on the transfer and utilization of dietary linoleic acid in lactating mothers demonstrated rapid transfer of acid to milk with only 3.5 to 4.5% of ingested FA being oxidized to CO_2_. This may suggest a low contribution of linoleic acid as an energy source^18^. It is thus possible that due to the critical function of PUFAs in infant growth and brain development, these fatty acids are spared primarily for milk synthesis.

Our results are based on the analysis of a single sample of breast milk taken at the time of midmorning feeding. Although we standardized milk collection time against possible diurnal changes in milk composition, future studies should include repeated collection of milk samples both during the day and within a period of at least several days.

The design of our study allowed for measuring cortisol response to a single and mild laboratory stressor, however, it remains unclear how this single stressor affected milk composition. When interpreting the results, we assumed that the level of cortisol produced in reaction to the cold pressor test represented the usual pattern of an individual’s response to any single stressor. This assumption was made based on the existing literature demonstrating fairly stable individual patterns of cortisol release in response to stressors^62^. Future studies should plan multiple milk sample collection to allow taking into account the delay between the stressor and milk synthesis.

Furthermore, we assessed maternal life changes retrospectively, which might be associated with recall bias. Since milk composition might be affected by both past and current stressors, future studies should also include more detailed methods to assess current psychosocial stress.

Finally, to fully assess the effect of stress on milk production, the overall milk volume released during a breastfeeding episode should be measured. Such measurements would help to precisely quantify macronutrients transferred to the infant and to infer about the developmental outcome associated with milk production under stress.

In summary, the results of our study suggest that both short- and long-term responsiveness to stress is associated with an altered composition of breast milk. While short-term consequences of higher stress responsiveness result in providing milk with higher energy value and lipid content in mothers under chronic stress, this trend is reversed. Together with the results of previous studies, our study advocates monitoring maternal psychological wellbeing during pregnancy and postpartum to promote breastfeeding, healthy infant nutrition, and development during the first months of life.

## Methods

### Subjects and protocol

A sample of 160 mothers together with their healthy, born on-term infants from the area of Wroclaw, South-Western Poland, took part in a study on the association between maternal stress and breast milk composition. Participants were recruited from the city population to meet the following inclusion criteria: (1) neither mothers nor infants suffered from metabolic diseases such as diabetes or thyroid diseases and from genetically inherited conditions; (2) infants were born from a single, uncomplicated pregnancy, with the appropriate birth weight for gestational age not lower than 2500 g; (3) infants were fed on demand, exclusively with breast milk.

During the first meeting scheduled when the children were approximately five months old, the mothers signed the informed consent and were instructed about all study procedures. Trained study assistants performed maternal anthropometric measurements. Mothers also received questionnaires to be filled in at home: (1) a general questionnaire about basic maternal demographics: age, place of birth, education, life, and economic satisfaction, marital status, reproductive history, and infant and maternal health; (2) Recent Life Changes Questionnaire^30^ collecting information about maternal stressors and (3) daily logs of food intake and breastfeeding times. These questionnaires were returned at the second meeting approximately one week later. At this meeting, infant anthropometric measurements and milk sample collection occurred. The cold-pressor test^31^ was also conducted to assess maternal hormonal response to a mild stressor. The study protocol was approved by the Bioethical Committee of Lower Silesian Medical Chamber in Wroclaw. All research methods were performed in accordance with relevant guidelines and regulations.

Complete data on breast milk composition, maternal stress, and anthropometrics were available for 153 mothers and their infants. Data from 3 obese mothers (BMI > 35) were excluded from the analysis due to possible effects of obesity on the primary outcome measures^32^. Furthermore, data from 3 mothers with extremely high Recent Life Changes Questionnaire scores (> 3 SD) were also excluded due to possible psychopathology.

### Maternal stress, diet, and anthropometrics

The number and intensity of maternal stressors during the six months preceding joining the study were assessed using the Recent Life Changes Questionnaire (RLCQ)^30,33^. This questionnaire lists 55 life events such as death of a family member, job loss, divorce, diseases, etc. grouped into the domains of health, work, home, family, and personal. In addition to listing the events, mothers were asked to evaluate the significance and difficulty in adjusting to each of these events on a scale from 1 to 100. The total individual Recent Life Changes (RLC) score was a sum of scores given to each of the events that happened during the recent period of 6 months before the mother joined the study and thus covered the late prenatal and the whole postnatal period of infant life.

The maternal diet was assessed using daily logs from three random weekdays including one from the weekend. Mothers noted each food item, including supplements consumed during the day together with the size or weight of the portion and the time of consumption. These daily logs were further used to calculate the average intake of fat, carbohydrates, protein (in grams), total energy (in kcal), and selected fatty acids (in grams) using the computer program Diet 5D developed by Polish National Food and Nutrition Institute.

Maternal and infant anthropometrics at the entrance were measured using standard methods (details in Supplementary Materials). Birth weight and length data were collected from Child Health Records presented by mothers during the study visit. All infant anthropometric data were z-scored using WHO database as a reference group^34^.

### Breast milk sample collection and analysis

Mothers collected milk samples to sterile containers using a Medela Symphony breast pump (Medela AG, Switzerland) under the supervision of the research assistants at the meeting room. To standardize milk collection time against possible diurnal changes in breast milk composition, samples were collected between the second and the third feeding episode of the day, where the first feeding was the one after which an infant showed daytime activity^35,36^. Because the fat content varies considerably during the same feeding^37^, mothers were instructed to pump from one breast until empty. Since no significant difference in milk composition was found between left and right breast^38^, the mothers were free to choose from which breast milk was collected.

Immediately after collection, milk samples were portioned into smaller containers (10 and 15 ml) for further analysis and stored at 4 °C (fatty acid composition analysis) or at -20 °C (general macronutrient analysis). Samples for fatty acid composition analysis were transported to the laboratory for fat extraction within a few hours after collection, while samples for general macronutrient composition analysis were transferred to -80 °C for later analysis.

Content of macronutrients (protein, fat, carbohydrates) and energy density of milk was analyzed by mid-infrared transmission spectroscopy using MIRIS Human Milk Analyser (MIRIS, Sweden) according to the manufacturer instruction (details in Supplementary Materials).

The fatty acid concentrations were determined in 15 ml of fresh samples. Total lipids were extracted using chloroform: methanol (2:1 v/v) and the extracts evaporated to dryness under nitrogen^39,40^. Samples of 1 μl of the solution were analyzed using gas chromatography method (Agilent Technologies 5973). The concentration of fatty acids (g in 100 g of fat) was determined based on the obtained peak areas and read from the standard curves prepared for the analyzed acids (details in Supplementary Materials). These concentrations were further used to calculate the total quantity (in grams) of any given fatty acid and group of acids in 100 ml of milk by using values of fat obtained from the analysis of macronutrients. The calculation was done based on a simple proportion. Finally, data on individual fatty acids were grouped into medium-chain fatty acids (MCFA), long-chain fatty acids (LCFA), long-chain saturated fatty acids (LCFA-S), long-chain monounsaturated fatty acids (MUFA) and long-chain polyunsaturated fatty acids (PUFA) according to classical definition.

### Cold pressor test, salivary sample collection, and analysis

The hand cold-pressor test (CPT)^31^ was performed to assess maternal physiological reactivity to a mild stressor. Women were asked to immerse the hand into ice water for one minute. None of them terminated test before the expected participation time. Four saliva samples to measure cortisol levels were taken (1) 10 min. before (Cort_0_); (2) a min. before (Cort_10_); (3) during the test (Cort_11_), and 4) 10 min. after the test (Cort_21_). The area under the curve (Cort_AUC_) for the levels measured in all saliva samples was calculated to include in further analysis.

Samples were collected into sterile 1 ml Eppendorf tubes and stored at -80 °C until the assay. After thawing and centrifugation (1500 g for 10 min), samples were tested for salivary C concentration using enzyme-linked immunosorbent assays (Salivary Cortisol ELISA, DRG Instruments GmbH, Germany) according to the manufacturers’ recommendations. The samples were analyzed in duplicate, and the average intra-assay coefficient of variation for cortisol was less than 4.5%.

### Statistical analysis

For the purpose of sample description, maternal and infant data were assigned to 2 groups differing in the number and intensity of recent life stressors. Low RLC group consisted of mothers with RLC score equal to or lower than the median (Mdn = 374.0), while the high RLC group consisted of mothers with RLC scores higher than median. Differences between these two groups regarding demographic data, anthropometrics, breast milk composition, and Cort indicies were tested using Student t-test whenever the data indicated normal distribution or Mann–Whitney test when the distribution of the data diverged from normal. Data on Cort indicies was log-transformed (logCortAUC) due to its non-normal distribution. Effect sizes were estimated as Cohen's d (Student t-test) or rank-biserial correlations (Mann–Whitney test).

Pearson and Spearman correlation and linear regression were used to test for the effect of recent life stressors and stress reactivity on maternal diet and nutritional status. The RLC score and the area under the curve for cortisol response to CPT were correlated against specific estimated dietary intake (protein, fat, carbohydrates, and energy) and maternal body fat.

Hierarchical regression models were used to test for the association between the composition of breast milk and life stressors. Since higher stress may lead to increased levels of cortisol, and cortisol was previously demonstrated to channel nutrients toward the production of milk^41,42^, both factors were included in the models.

In the first step of each analysis, common factors such as child age, child BMI, the average number of feedings during the day, maternal body fat, and dietary intake of the corresponding milk component were included in the null model. These factors were demonstrated to predict milk composition in previous research. In the second step, postnatal RLC, together with logCort_AUC_ were included in the final model. All analyses were conducted using JASP 0.10.2 (JASP Team, 2019).

## Supplementary Information

Supplementary Information.
