# Supplementary material for: Psychosocial stress and cortisol stress reactivity predict breast milk composition
Source: Sci Rep. 2021 Jun 2;11:11576. doi: 10.1038/s41598-021-90980-3 (PMC8172899; doi:10.1038/s41598-021-90980-3)
Supplement: Supplementary file 1 — Supplementary Information. [file 41598_2021_90980_MOESM1_ESM.pdf]

Psychosocial stress and cortisol stress reactivity predict breast milk composition

Anna Ziomkiewicz* [1, 2], Magdalena Babiszewska [2], Anna Apanasewicz [2], Magdalena Piosek [3], Patrycja Wychowaniec [4], Agnieszka Cierniak [5], Olga Barbarska [6], Marek Szołtysik [7], Dariusz Danel[2], Szymon Wichary [8]

1. Institute of Zoology and Biomedical Sciences, Jagiellonian University, Gronostajowa 9, 30-387, Kraków, Poland

2. Ludwik Hirszfeld Institute of Immunology and Experimental Therapy, Polish Academy of Sciences, Rudolfa Weigla 12, 53-114 Wroclaw, Poland

3. Institute of Psychology, University of Wroclaw, J.W. Dawida 1, 50-527 Wroclaw, Poland

4. Faculty of Biology, Department of Human Biology, University of Wroclaw, Szewska 48, 50-139 Wroclaw, Poland

5. Faculty of Medicine and Health Sciences, Andrzej Frycz Modrzewski Krakow University,

Gustawa Herlinga-Grudzinskiego 1, 30-705 Krakow, Poland

6. Laboratory of Human Milk and Lactation Research at Regional Human Milk Bank in Holy Family Hospital, Faculty of Health Sciences, Medical University of Warsaw, Zwirki i Wigury 63a, 02-091 Warsaw, Poland

7. Faculty of Biotechnology and Food Science, Wroclaw University of Environmental and Life Sciences, J. Chelmonskiego 37, 51-630 Wroclaw

8. Institute of Psychology, Jagiellonian University, Ingardena 6, 30-060 Krakow, Poland

*anna.ziomkiewicz-wichary@uj.edu.plSupplementary Materials

Maternal and infant anthropometrics

Maternal height was measured using a stadiometer. Weight and body fat were measured with Tanita SC-240 MA Body Scale (Tanita Corp., Japan). Infant weight was measured to the nearest 1 g by using a hospital analog infant scale. The supine length was measured to the nearest 0.1 cm using an infant measuring board (Seca GmbH, Germany).

Breast milk sample analysis

Content of macronutrients

Milk samples of 10 ml were slowly warmed to 40 °C before testing and homogenized with MIRIS Sonicator (an ultrasonic homogenizer) (1.5 s/1 ml). Each sample was tested three times, using 3 ml of milk per one replication, and the average from 3 measurements was reported.

Content of fatty acids

Separation was carried out under the following conditions: column HP-88 (100m x 0,250 mm x 0.20 μm), column temperature from 70˚C (5min) to 240˚C (4˚C/min), the carrier gas - helium (20 cm/s), injection spray 1μl, 260˚C, split 100:1. DHA and AA were identified based on retention times obtained for standards and by comparing their mass spectra with the standards.

Salivary sample analysis

Enzyme-linked immunosorbent assays is a solid phase based on the principle of competitive binding. The optical density was determined with a microplate reader (LEDETECT96, Labexim Products, Austria) at 450 nm. The sensitivity provided by the manufacturer is 0,09 ng/ml, with a detection range of 0,09 - 30 ng/ml. All the analyzed samples felt into this range.
